# Improvement of Electrical and Thermal Properties of Carbon Nanotube Sheets by Adding Silver Nanowire and Mxene for an Electromagnetic-Interference-Shielding Property Study

**DOI:** 10.3390/nano14191587

**Published:** 2024-10-01

**Authors:** Matthew Kurilich, Jin Gyu Park, Joshua Degraff, Qiang Wu, Richard Liang

**Affiliations:** 1 High-Performance Materials Institute (HPMI), Florida State University, Tallahassee, FL 32310, USA; 2 Department of Materials Science and Engineering, FAMU-FSU College of Engineering, Tallahassee, FL 32310, USA; 3 Department of Industrial and Manufacturing Engineering, FAMU-FSU College of Engineering, Tallahassee, FL 32310, USA

**Keywords:** carbon nanotubes, silver nanowire, MXene, EMI shielding, transport properties

## Abstract

Hybrid carbon nanotube (CNT) sheets were fabricated by mixing CNTs with silver nanowires (AgNWs) and MXene to study their electromagnetic-interference (EMI)-shielding properties. CNT/AgNW and CNT/MXene hybrid sheets were produced by ultrasonic homogenization and vacuum filtration, resulting in free-standing CNT sheets. Three different weight ratios of AgNW and MXene were added to the CNT dispersions to produce hybrid CNT sheets. Microstructure characterization was performed using scanning electron microscopy, and the Wiedemann–Franz law was used to characterize transport properties. The resulting hybrid sheets exhibited improved electrical conductivity, thermal conductivity, and EMI-shielding effectiveness compared to pristine CNT sheets. X-band EMI-shielding effectiveness improved by over 200%, while electrical conductivity improved by more than 1500% in the hybrid sheets due to a higher charge-carrier density and synergistic effects between nanomaterials. The addition of AgNW to CNT sheets resulted in a large improvement in electrical conductivity and EMI shielding; however, this may also result in increased weight and sample thickness. Similarly, the addition of MXene to CNT sheets may result in an increase in weight due to the presence of the denser MXene flakes.

## 1. Introduction

Modern communication technologies, such as cell phones and radios, rely on devices to accurately transmit and receive electromagnetic waves (EMWs) to exchange information. Protecting these devices from harmful EMWs that may disrupt communications is critical. The disruption of electronics by their interaction with EMWs is called electromagnetic interference (EMI) and can lead to the misinterpretation of data and, in some cases, device failure [1]. Shielding materials block EMI through two primary mechanisms: reflection and absorption. The reflection of the EMW occurs due to an impedance difference between the two mediums in which the wave must travel; here, air is a high-impedance medium, and as the impedance of the shield is lower, the more electrically conductive it is. Inside the shield, the EMW is absorbed due to generated eddy currents, which is also dependent on electrical conductivity as well as magnetic permeability [2].

This research aims to introduce tunable lightweight materials that can enhance EMI-shielding performance. Shielding materials are currently primarily metals due to their high electrical conductivity and manufacturability [3]. Due to the high density of metals, recent research trends in industries, such as wearable electronics, are seeking to replace metals in high-performance applications with lightweight materials that decrease weight in order to encourage low-profile electronics and improve maneuverability [4,5,6]. Additionally, new EMI-shielding materials must also demonstrate sufficient thermal conductivity to effectively dissipate heat. As integrating EMI shielding into systems with high-frequency electronics becomes commonplace, these electronics produce heat and are susceptible to overheating [7].

Since the discovery of carbon nanotubes (CNTs) in 1991, they have been widely investigated for their mechanical, electrical, and thermal properties [8,9,10]. CNTs are lightweight and can be assembled into large sheets and roll-to-roll products through dispersion techniques. Given the multifunctionality and low density of CNT-based materials, they have become attractive potential candidates as lightweight EMI-shielding materials [11,12,13,14]. Additionally, CNTs have excellent thermal transfer properties, making them an attractive candidate for board-level shielding applications [15].

MXenes are a new class of 2D materials produced from a MAX phase material, which consists of a transition metal (M), aluminum (A), and either carbon, nitrogen, or a combination of the two elements (X). To produce MXene from the MAX phase, aluminum is removed via selective etching, and the remaining material is exfoliated to produce thin 2D flakes. The resulting metal carbonitride layers exhibit excellent electrical conductivity for a myriad of next-generation EMI-shielding applications [16,17].

Silver nanowires (AgNWs) are another attractive material for EMI-shielding applications due to their high intrinsic electrical conductivity, large aspect ratio, and ease of manufacturing [18,19]. AgNW’s large aspect ratio make it an ideal filler as it can percolate into a network at low concentrations and can be added to many polymer matrices, as well as CNTs, to form sheets for EMI-shielding applications [20,21]. 

CNTs, MXenes, and silver nanowires (AgNWs) have each been studied as potential EMI-shielding materials [22,23,24]. In addition, hybrid composites of CNT/AgNWs and CNT/MXenes have been extensively studied to achieve improved properties compared to CNTs, AgNWs, and MXenes individually [20,25,26,27,28,29,30,31,32]. Zhang et al., Jing et al., and Choi et al. reported improved electrical properties by as much as 10,000% for CNT/AgNW hybrid composites [20,26,27]. Oluwalowo et al. also reported improved electrical conductivity and thermal properties as a result of the synergistic effects between AgNWs and CNTs [28]. Wang et al. fabricated CNT/AgNW sandwich structures that achieved an SE as high as 72 dB [21]. Zhao et al. reported conductivity as high as 385 S cm^−1^ for layered CNT/MXene hybrid papers [29]. Liang et al. demonstrated that CNTs help prevent MXene layers from restacking and ensure that the MXene layers maintain a 2D morphology, resulting in improved conductivity in the hybrid material [30]. Yang et al. report an improvement in EMI-shielding properties in CNT sheets via the addition of MXene, achieving an SE as high as 60.5 dB in the X-band [31]. Xue et al. fabricated CNT/MXene/polyimide aerogels that had an average SE of 68.2 dB with low reflection [32]. The literature indicates that the combination of CNTs with either MXene or AgNW leads to heightened EMI-shielding, thermal, and electrical properties. Furthermore, the addition of nanomaterials to fiber-reinforced materials may lead to additional multifunctional properties, including flame-retardancy and improved mechanical properties [33,34].

Although many reports exist that discuss improvement in properties of CNT hybrid sheets, few report comparisons between different hybrid sheets follow the same manufacturing procedure. Additionally, few reports exist that characterize transport properties in CNT/AgNW and CNT/MXene hybrid sheets. In this article, we report in our study on the improvements in the electrical conductivity, thermal conductivity, and EMI-shielding performance of CNT hybrid sheets with multiple concentrations of MXene and AgNW produced by the same ultrasonic-dispersion and vacuum-filtration processes. The Wiedemann–Franz law was used to characterize phonon and electron transport within CNT/AgNW and CNT/MXene hybrid sheets. Scanning electron microscopy (SEM) was used to characterize sample morphology, and a structure–property–performance relationship was established.

## 2. Methods

### 2.1. Materials

The CNTs used in this research were vertically aligned multi-walled CNTs (MWCNTs) 1–2 mm long and 20 nm in diameter, purchased from General Nano (Cincinnati, OH, USA). AgNWs were used as received from Advanced Chemical Supplier (Pasadena, CA, USA) in the form of an aqueous dispersion. The length and diameter of the AgNWs were 100–200 µm and 50 nm, respectively. Ti_3_C_2_T_x_ MXene was produced in-house by selectively etching MAX phase material purchased from American Elements (Los Angeles, CA, USA). The etching of aluminum was performed using LiF with HCl, and the intercalation was performed using TMAOH before centrifuging to isolate single and few-layer MXene flakes. 

### 2.2. Fabrication of Hybrid CNT Sheets

**Figure 1:**
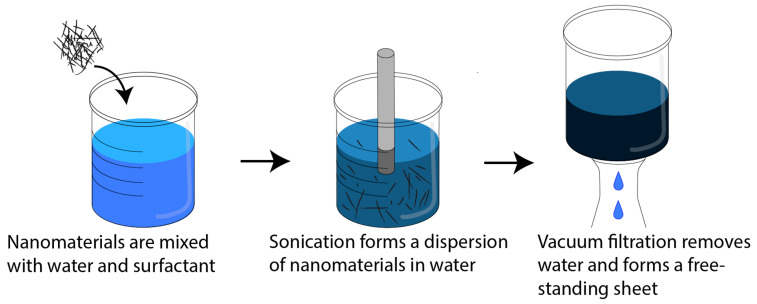
Ultrasonic dispersion and filtration processes.

CNT/AgNW and CNT/MXene hybrid nanotube sheets were fabricated by ultrasonic dispersion and vacuum filtration, as shown in Figure 1. Due to van der Waals forces among individual CNTs, they are difficult to disperse in pure water without surface modifications [35]. As a result, the CNTs tend to agglomerate and fall out of solution, leading to low-quality films [36]. The addition of a surfactant, such as Triton X-100, can de-bundle and stabilize individual nanotubes, allowing CNTs to form homogenous aqueous dispersions [37]. To produce the hybrid sheets, CNTs were added to 200 mL of DI water along with 2 mL of Triton X-100. The CNTs were then dispersed by a QSonica Q700 tip sonicator for 45 min. To preserve the lengths of the CNTs and prevent excessive heating, sonication was performed in 10 s pulses within an ice bath. This method for dispersing CNTs in solution was previously characterized and shows results in well-dispersed suspensions, as characterized by UV-Vis [38]. Following CNT dispersion, either AgNW or MXene was then stirred into the CNT dispersion, followed by an additional sonication time of 5 min. The dispersion was immediately filtered via vacuum-assisted filtration through a nylon filter with a pore size of 0.45 µm. The resulting free-standing papers were allowed to dry overnight before being washed 5 times with isopropyl alcohol to remove any residual surfactant. A TA Instruments TGA Q50 was used to confirm the removal of the surfactant via thermogravimetric analysis (TGA) at 10 °C per minute up to 900 °C in air. TGA data can be found in the Supplementary Material, Figure S1. The removal of the surfactant is critical for maximizing the electrical properties of the resultant CNT sheet. Table 1 lists the samples fabricated for this study, including the type of nanomaterial, weight percent, and volume percent added to the CNTs. The samples are named based on the weight percent and type of nanomaterial filler (i.e., 50C50A contains 50 wt% CNT and 50 wt% AgNW). Each sample was made using the same total weight of material so that each hybrid paper had the same areal density regardless of paper density and thickness. 

### 2.3. Hybrid Sheet Structure Characterization

The structure of CNT/AgNW and CNT/MXene hybrid sheets was characterized by SEM and X-ray diffraction (XRD). Sample morphology was studied via SEM using a Thermo-Fisher (Waltham, MA, USA) FEI Helios G4 dual beam microscope. Crystalline structures in the hybrid sheets were measured using a Rigaku (The Woodlands, TX, USA) Smartlab Powder X-ray diffractometer. XRD results are given in the Supplementary Material, Figure S2. 

### 2.4. Measurement of Electrical Properties and EMI Shielding

**Figure 2:**
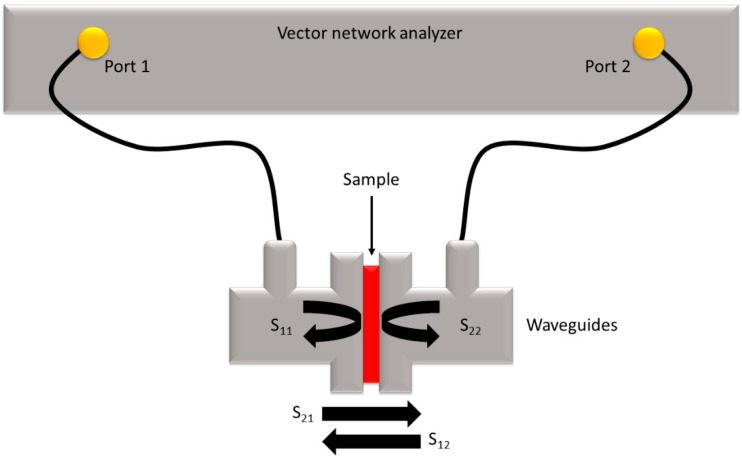
Network analyzer and scattering parameters.

Electrical conductivity was measured using a Suragus Eddycus TF Lab 2020. EMI-shielding effectiveness (SE) was measured using a Keysight (Santa Rosa, CA, USA) M937A vector network analyzer (VNA) and WR90 waveguides in the X-band range (8.2–12.5 GHz). Figure 2 presents a diagram of the experimental setup that includes the VNA, waveguides, and the four scattering parameters (S-parameters). These S-parameters are labeled S_ab_, where a and b denote the ports that receive and send the signal, respectively. S_11_ and S_22_ represent the reflection of incident signals, while S_12_ and S_21_ represent the transmission. The scattering parameters S_11_ and S_12_ were used to calculate the reflection and absorption components of shielding effectiveness. 

### 2.5. Measurement of Thermal Properties

In-plane thermal diffusivity was measured at room temperature via laser flash analysis (LFA, Netzsch 457 Microflash, Burlington, MA, USA). The mean diffusivity was determined after three runs. Modular differential scanning calorimetry (MDSC) was used to measure specific heat at room temperature. Equation (1) was used to calculate thermal conductivity (κ), where α denotes the thermal diffusivity; ρ denotes the bulk density; and C_p_ denotes the specific heat capacity.

(1) $$\kappa =\alpha \rho C_{p}$$

## 3. Results and Discussion

### 3.1. Microstructure Analysis

**Figure 3:**
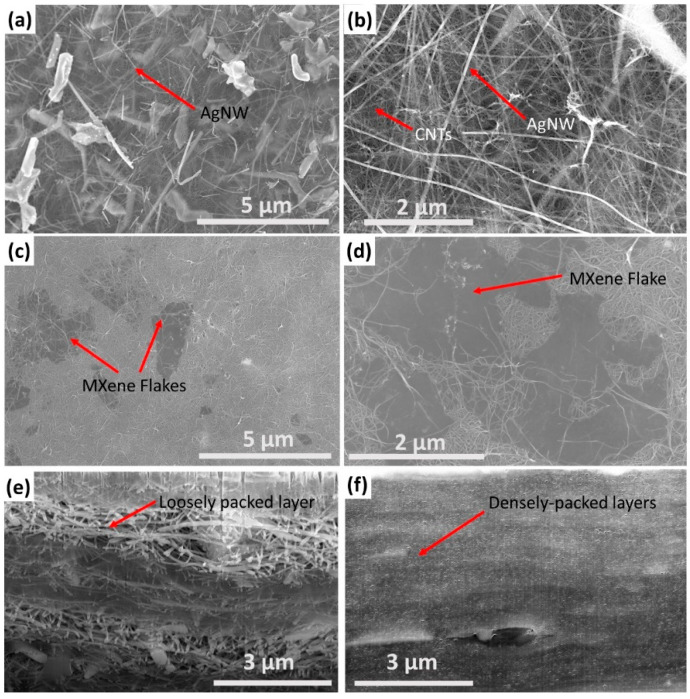
Morphology of CNT hybrid sheets containing 50 wt% filler. SEM images of the following (**a**): (**b**) CNT/AgNW hybrid sheet. (**c**,**d**) CNT/MXene sheet. (**e**) Cross-section of CNT/AgNW hybrid sheet. (**f**) Cross-section of CNT/Mxene hybrid sheet.

Figure 3a,b present SEM images of the surface of 50 wt% CNT/AgNW hybrid sheets. Individual NWs can be seen dispersed throughout the CNT network and appear to form a fully percolated network of their own at this loading, suggesting a sharp increase in properties at 50 wt%. Figure 3c,d present SEM images of the surface of 50 wt% CNT/MXene hybrid sheets. MXene flakes of less than ~1 µm and up to ~5 µm are visible both on and within the CNT networks. CNTs can be faintly seen through the MXene layers, confirming their few-layer structure. Here, the MXene flakes are seen to be disconnected from one another, suggesting that the high loading of MXene may be necessary to result in a more electrically conductive network. Figure 3e,f display the cross-section of CNT/AgNW and CNT/MXene hybrid sheets, respectively. The addition of AgNW resulted in a loosely packed network, as shown in Figure 3e. Contacts among CNTs and AgNWs can form a variety of nanostructures, which has been discussed in the literature. [26,39]. At 50 wt%, the addition of MXene results in a dense network due to the restacking of individual 2D layers. This dense network reduces sample thickness but may also cover MXene active sites and reduce charge carrier mobility [40]. Additionally, in both cases, some separation of the constituent nanomaterials can be seen, which may be caused by uneven settling during the vacuum-filtration process. 

### 3.2. Electrothermal Analysis

**Figure 4:**
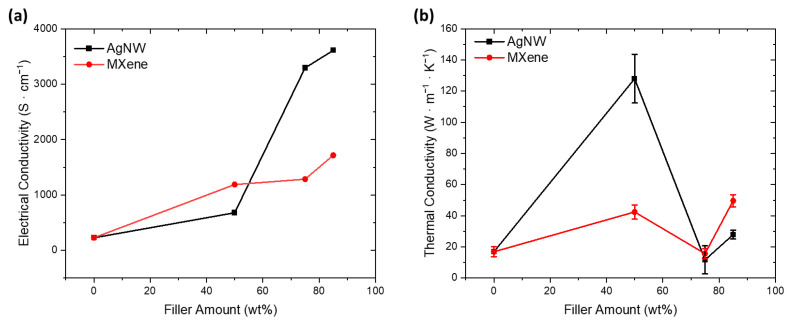
Change in (**a**) electrical and (**b**) thermal conductivity with increasing filler weight.

As expected, the addition of AgNW or MXene into CNT sheets resulted in an increase in electrical conductivity [27,30]. Figure 4a displays these results. The improved electrical conductivity can be attributed to synergistic interactions among the nanomaterials in addition to the additives possessing higher conductivity than CNTs, as AgNW and MXene possess an electrical conductivity of 6.3 × 10^9^ S∙cm^−1^ and 2 × 10^4^ S∙cm^−1^, respectively, while the CNTs used in this study have a conductivity of 227 S∙cm^−1^ [25,30,41,42]. AgNW and MXene have charge-carrier densities of 5.85 × 10^22^ cm^−3^ and ~2 × 10^21^ cm^−3^, respectively, while the density of charge carriers in MWCNTs is in the order of 9 × 10^20^ cm^−3^ [43,44,45].

As the concentration of AgNW exceeded 50 wt%, there was a drastic increase in electrical conductivity; however, this was not the case for MXene. As can be seen in Figure 3a, AgNWs were able to easily make contact across the sample due to the high aspect ratio of the wires. However, Figure 3c shows disconnected MXene flakes within the CNT network, which contribute little toward increasing conductivity. 

Figure 4b presents the thermal conductivity of CNT hybrid sheets with respect to increasing the filler content. The thermal conductivity of pristine CNTs was 16.88 W·m^−1^·K^−1^, which is in line with the literature results [46]. In both CNT/AgNW and CNT/MXene hybrid sheets, 50 wt% filler resulted in improved thermal conductivity due to the presence of more thermally conductive nanomaterials. However, the thermal conductivity drops at high concentrations of both AgNW and MXene, potentially due to the uneven filtering of the nanomaterials, as can be seen in SEM. Higher concentrations of AgNW or MXene may lead to more uneven filtering and, hence, a worse dispersion, giving these samples lower thermal conductivity. The effects of filler content on thermal conductivity (κ) were similar to electrical conductivity (σ), as there exists a relationship between the two properties described by the Wiedemann–Franz law, presented in Equation (2).

(2) $$\frac{\kappa }{\sigma }=LT$$

In this equation, T is the temperature in Kelvin, and the constant of proportionality, L, is called the Lorenz number. In metals, the Lorenz number approaches the Sommerfeld value, L_0_ = 2.45 × 10^−8^ W·Ω·K^−2^ [47]. The Lorenz number tends to vary from this Sommerfeld value with respect to the ratio of the mean free path of thermal conduction ($l_{\tau })\;$to the mean free path of electrical conduction ($l_{\varepsilon }$), as presented in Equation (3) [48].

(3) $$L=L_{0}\left(\frac{l_{\tau }}{l_{\varepsilon }}\right)$$

**Figure 5:**
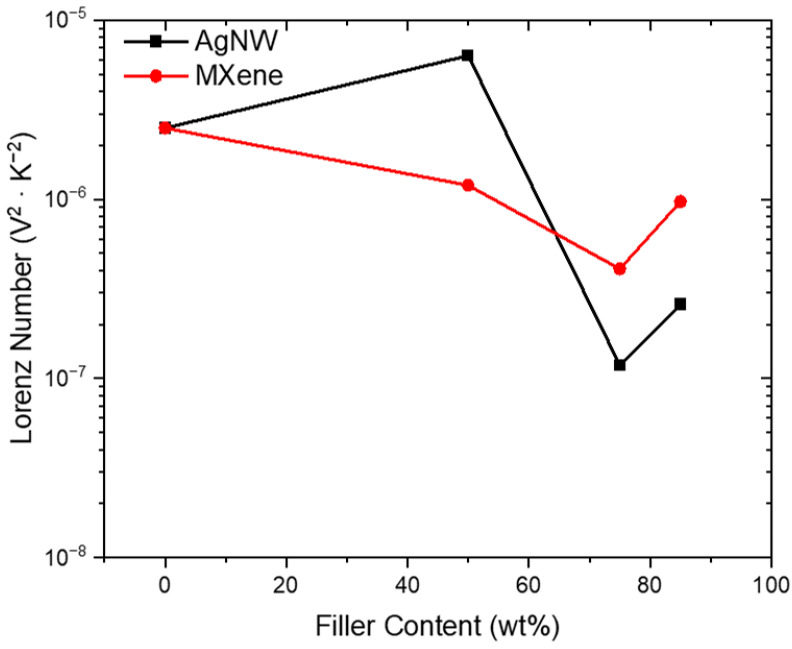
The calculated Lorenz number for CNT/AgNW hybrid sheets and CNT/MXene hybrid sheets at 298 K.

Figure 5 plots the Lorenz numbers of CNT/AgNW and CNT/MXene hybrid sheets. The Lorenz number for the pristine CNT sheet was found to be 2.5 × 10^−6^ V^2^·K^−2^, which indicates that the mean free path for thermal conduction was significantly higher than the mean free path for electrical conduction. This suggests the existence of phonon-dominant thermal conduction in CNTs, as has been previously reported [46]. In general, the addition of nanomaterial fillers to CNT reduced the Lorenz number, which brought it closer to the Sommerfeld value and more in accordance with metallic conduction. The 25C75A sample exhibited the highest degree of electron thermal conduction with a Lorenz number of 7.19 × 10^−8^ V^2^·K^−2^. For both AgNW and MXene, increasing the filler content from 75 wt% to 85 wt% appeared to show an increase in Lorenz value. 

At 75 wt%, the more electrically conductive materials were fully percolated through the CNT network, creating a robust electrically conductive network across the hybrid sheet. The further addition of either AgNW or MXene did not drastically improve electrical conductivity; however, improvements to thermal conductivity were observed due to fewer boundaries on which phonons could scatter [49]. Table 2 summarizes the electrical and thermal conductivity results.

### 3.3. EMI-Shielding Properties

When an electromagnetic wave interacts with a conductor, charges within the conductor move to cancel the electric field, while generated eddy currents cancel the applied magnetic field inside the shield. Therefore, the most important properties of the shielding material are electrical conductivity, magnetic permeability, and shield thickness. Equations (4)–(6) express the critical properties and interactions that influence plane-wave shielding [50].

(4) $${SE}_{R}=10\log_{10}\left(\frac{\sigma }{16\omega \varepsilon_{0}\mu }\right)$$

(5) $${SE}_{A}=10\log_{10}\left(e^{t\sqrt{\omega \mu \sigma /2}}\right)$$

(6) $${SE}_{T}\left(dB\right)={SE}_{R}+{SE}_{A}=10\log_{10}\left(\frac{\sigma }{16\omega \varepsilon_{0}\mu }\right)+10\log_{10}\left(e^{t\sqrt{\omega \mu \sigma /2}}\right)$$

**Figure 6:**
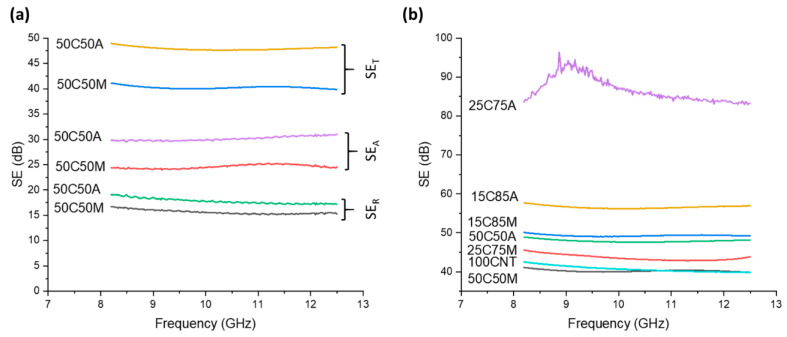
EMI-shielding properties: (**a**) reflection, absorption, and total SE for pristine CNT, 50 wt% CNT/AgNW, and CNT/MXene hybrid sheets. (**b**) SE of the pristine and hybrid sheet.

In these equations, σ denotes electrical conductivity (S·m^−1^); ω denotes frequency (Hz); ε_0_ is the electrical permittivity of air (F·m^−1^); µ denotes the magnetic permeability of the shielding material (H·m^−1^); and t denotes the thickness of the shield (m). Even at low filler concentrations of AgNW and MXene, the hybrid sheets improved shielding—especially reflective shielding. This improvement is attributed to the combination of high conductivity and low thickness in the CNT hybrid sheets. Figure 6a reveals the SE_R_, SE_A_, and SE_T_ of the hybrid sheets containing 50 wt% filler. SE_A_, SE_A_, and SE_T_ of the hybrid sheets containing higher filler concentrations can be found in the Supplementary Material, Figure S3. In the case of all hybrid sheets, the shielding mechanism was dominated by reflection, with up to 99% of the contribution coming from reflection. This was due to the hybrid sheets having very high electrical conductivity and low thickness. Figure 6b shows the total SE of all samples in this study, where 25C75A showed the highest SE, achieving an over 90 dB reduction at 9 GHz. Interestingly, 50C50M showed a lower SE than pristine CNT despite its higher electrical conductivity. This is attributed to a decreased thickness in the CNT/MXene hybrid. Normalizing the SE by density and thickness provides further insight into the interactions between nanomaterials. When normalized by density and thickness, 50C50M shows the highest SE, followed by 25C75M. This is attributed to the fact that MXene has a much lower density than silver, and thus, the addition of low concentrations may greatly increase SE with minimal impact on density. 

**Figure 7:**
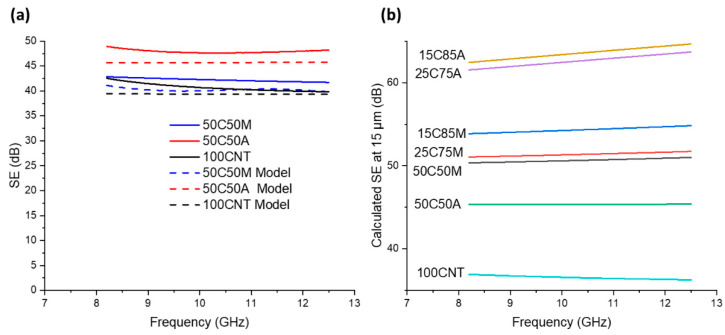
(**a**) Comparison of the modeling results to measured results for pristine and 50 wt% CNT/AgNW and CNT/MXene sheets. (**b**) Modeling results of pristine and hybrid sheets with 15 µm thickness.

In some applications, thickness may be a more important factor than areal density, and so Equations (4)–(6) can be deployed to model and compare the SE of each film independent of thickness. Figure 7a compares the measured and calculated SE values for 100CNT, 50C50A, and 50C50M samples. Electrical conductivity and sample thickness were directly measured, with magnetic permeability assumed to be ~µ_0_. The model agreed with the measured results to within ~3 dB. This indicates that the SE performance can be accurately estimated using the established equations. Figure 7b displays the SE predictions for pristine CNT sheets and each hybrid CNT sheet at a thickness of 15 µm. Here, electrical conductivity and magnetic permeability were the only variables. The addition of a small amount of AgNW (50 wt%) resulted in relatively small improvements in SE while increasing the concentration to 75 wt% resulted in much higher shielding. At loadings higher than 75 wt%, AgNW appeared to have diminishing returns. 

Conversely, the addition of 50 wt% MXene resulted in a 74% improvement in shielding effectiveness compared to pristine CNT sheets. At 8.2 GHz, SE improved from 37 dB to 50 dB. Increasing the MXene content beyond 50 wt% yields small gains in SE, as SE improves from 50 dB to 54 dB at 85 wt%. 

## 4. Conclusions

Dispersion and vacuum-filtration procedures were used to form CNT hybrid sheets containing high-weight percentages of AgNW and Ti_3_C_2_T_x_ MXene. The addition of 1D and 2D nanomaterials to CNT sheets resulted in increased electrical and thermal conductivity due to the increased number of conductive paths and positive synergistic effects between the nanomaterials. The hybrid sheets also showed increased EMI shielding at different filler contents, but EMI shielding and density must be optimized for lightweight applications. Additionally, the resulting hybrid sheets also show phonon-dominant thermal conduction, as calculated by the Wiedemann–Franz law. The results indicate that the addition of AgNW or MXene to CNT sheets results in improved electrical and thermal conductivity and higher EMI shielding across the X-band. The addition of AgNW to CNT sheets resulted in only small improvements at low concentrations, while the addition of 75 wt% AgNW showed vast improvements in SE over the pristine CNT sheet. AgNW greatly improved the electrical conductivity of CNT sheets, resulting in an increased SE in the X-band. However, this also increased the sample weight, and CNT/AgNW hybrid sheets showed a lower SE than CNT/MXene hybrids when normalized by thickness and density. Small additions of MXene to CNT resulted in greater improvements in SE, and increasing the MXene content further showed only small increases.

## Figures and Tables

**Table 1:** Sample naming convention, filler amount in weight, and volume percent.

Sample Name	Filler (wt%)	Filler (vol%)
100C	0%	0%
50C50A	50% AgNW	6.25% AgNW
25C75A	75% AgNW	9.1% AgNW
15C85A	85% AgNW	10.19% AgNW
50C50M	50% MXene	15.91% MXene
25C75M	75% MXene	22.10% MXene
15C85M	85% MXene	24.34% MXene

**Table 2:** Summary of electrical and thermal conductivity for CNT/AgNW and CNT/MXene hybrid sheets at 298 K.

Sample	Electrical Conductivity (S·cm^−1^)	Thermal Conductivity (W·m^−1^·K^−1^)
100C	227	16.9 ± 3.1
50C50A	679	128.0 ± 15.7
25C75A	3294	11.7 ± 8.9
15C85A	3610	27.9 ± 2.9
50C50M	1189	42.4 ± 4.5
25C75M	1285	15.6 ± 3.2
15C85M	1717	49.7 ± 3.9

## Data Availability

Data are contained within the article and Supplementary Materials.

## Supplementary Materials

The following supporting information can be downloaded at: https://www.mdpi.com/article/10.3390/nano14191587/s1, Figure S1: TGA curves of unwashed CNT sheet, washed CNT sheet, and hybrid CNT sheets containing 50 wt% AgNW and 50 wt% MXene; Figure S2: XRD of CNTs used in this study, commercially available CNTs, and (a) AgNW and 50C50A, and (b) MXene and 50C50M.
