# Supplementary material for: Improvement of Electrical and Thermal Properties of Carbon Nanotube Sheets by Adding Silver Nanowire and Mxene for an Electromagnetic-Interference-Shielding Property Study
Source: Nanomaterials (Basel). 2024 Oct 1;14(19):1587. doi: 10.3390/nano14191587 (PMC11478732; doi:10.3390/nano14191587)
Supplement: Supplementary file 1 [file nanomaterials-14-01587-s001.zip › nanomaterials-3192555-supplementary.pdf]

Supplementary Material For

Improvement of Electrical and Thermal Properties of Carbon Nanotube Sheets by Adding Silver Nanowire and MXene for Electromagnetic Interference Shielding Property Study

Matthew Kurilich [1,2], Jin Gyu Park [1,3], Joshua Degraff [1,3], Qiang Wu [1,3] and *Richard Liang [1,3]

[1] High-Performance Materials Institute (HPMI), Florida State University, Tallahassee, FL, 32310, USA
[2] Department of Materials Science and Engineering, FAMU-FSU College of Engineering, Tallahassee, FL, 32310, USA
[3] Department of Industrial and Manufacturing Engineering, FAMU-FSU College of Engineering, Tallahassee, FL, 32310, USA
* Correspondence: Liang@eng.famu.fsu.edu (R. L.)

1. Thermogravimetric Analysis (TGA)

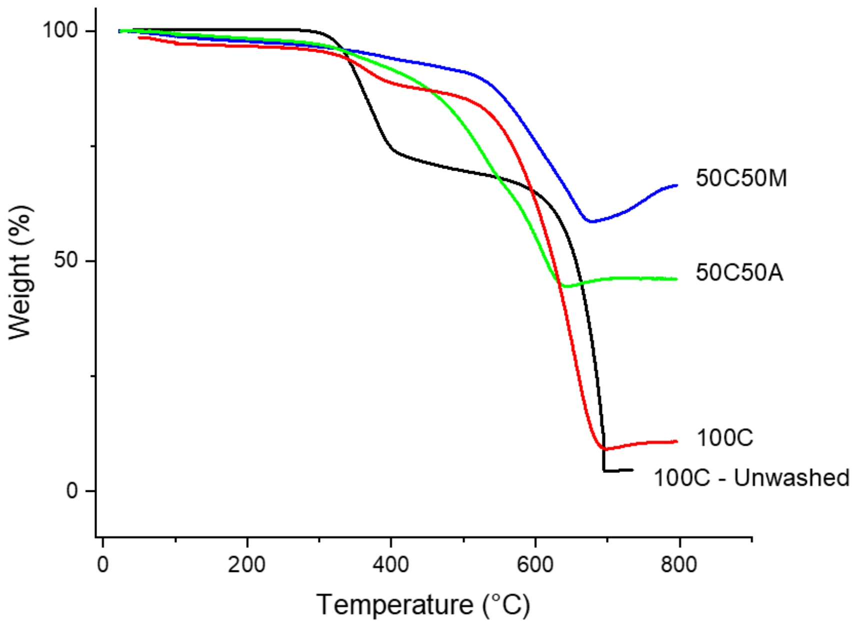

Figure S1. TGA curves of unwashed CNT sheet, washed CNT sheet, and hybrid CNT sheets containing 50 wt% AgNW and 50 wt% MXene.

TGA was used to characterize removal of surfactant from the hybrid sheets, as shown in Figure S1. In the unwashed CNT sheet, a drop in weight of ~25% can be seen between 350° and 400° and is associated with the surfactant burning off. By 700 °C, all of the CNTs have burned off as well and between 10-20 wt% remains, which is residual iron catalyst particles. In the washed CNT sample, there appears to be significantly less surfactant, and the sample loses around 8 wt% at 350 °C while showing a similar amount of remaining iron catalyst. In 50C50A, there appears to be no residual surfactant, although the CNT appears to burn-off at a slightly lower temperature. 50C50M shows similar behavior to 50C50A, except that it shows a large increase in weight at higher temperatures due to oxidation of the MXene flakes.

* Liang@eng.famu.fsu.edu

2. X-Ray Diffraction

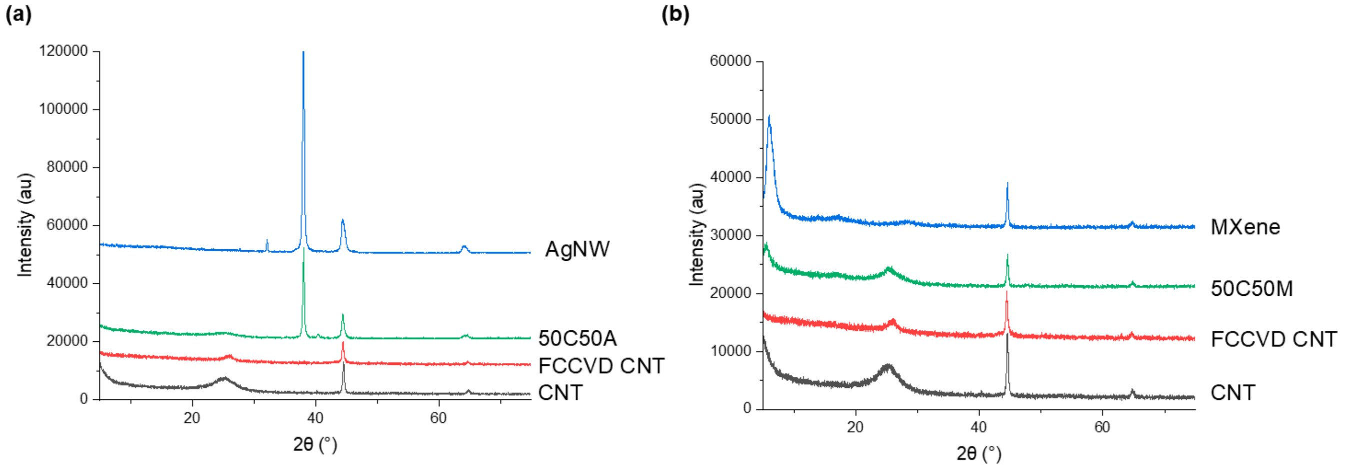

Figure S2. XRD of CNTs used in this study, commercially available CNTs, and (a) AgNW and 50C50A, and (b) MXene and 50C50M.

Figure S2 shows the results of XRD performed on the pristine CNT, MXene, and AgNW in this study as well as the CNT hybrid sheets containing 50 wt% AgNW and 50 wt% MXene. XRD of a commercially available CNT sheet (Nanocomp, United States) produced via floating catalyst chemical vapor deposition (FCCVD) was measured to serve as a comparison for the pristine CNT sheet used in this study. In the case of both our CNT sheet and the commercially available CNT sheet, a 26° peak associated with carbon and a 45° peak associated with the iron catalyst particle can be easily seen. Figure S2(a) shows pristine AgNW, where the peaks associated with silver at 38° (110), 44° (200), and 64° (220) can be easily seen. In 50C50A, the silver peaks remain easily visible, although the 26° carbon is very small. In Figure S2(b) the 9.7° (002) peak of MXene can be seen, and its slightly broad shape indicates the presence of a small amount of poorly exfoliated MXene sheets. The 19.1° (004) peak can be seen as well. Similar to 50C50A, 50C50M shows characteristic peaks of both constituent materials.

3. Shielding Mechanism of Hybrid Sheets

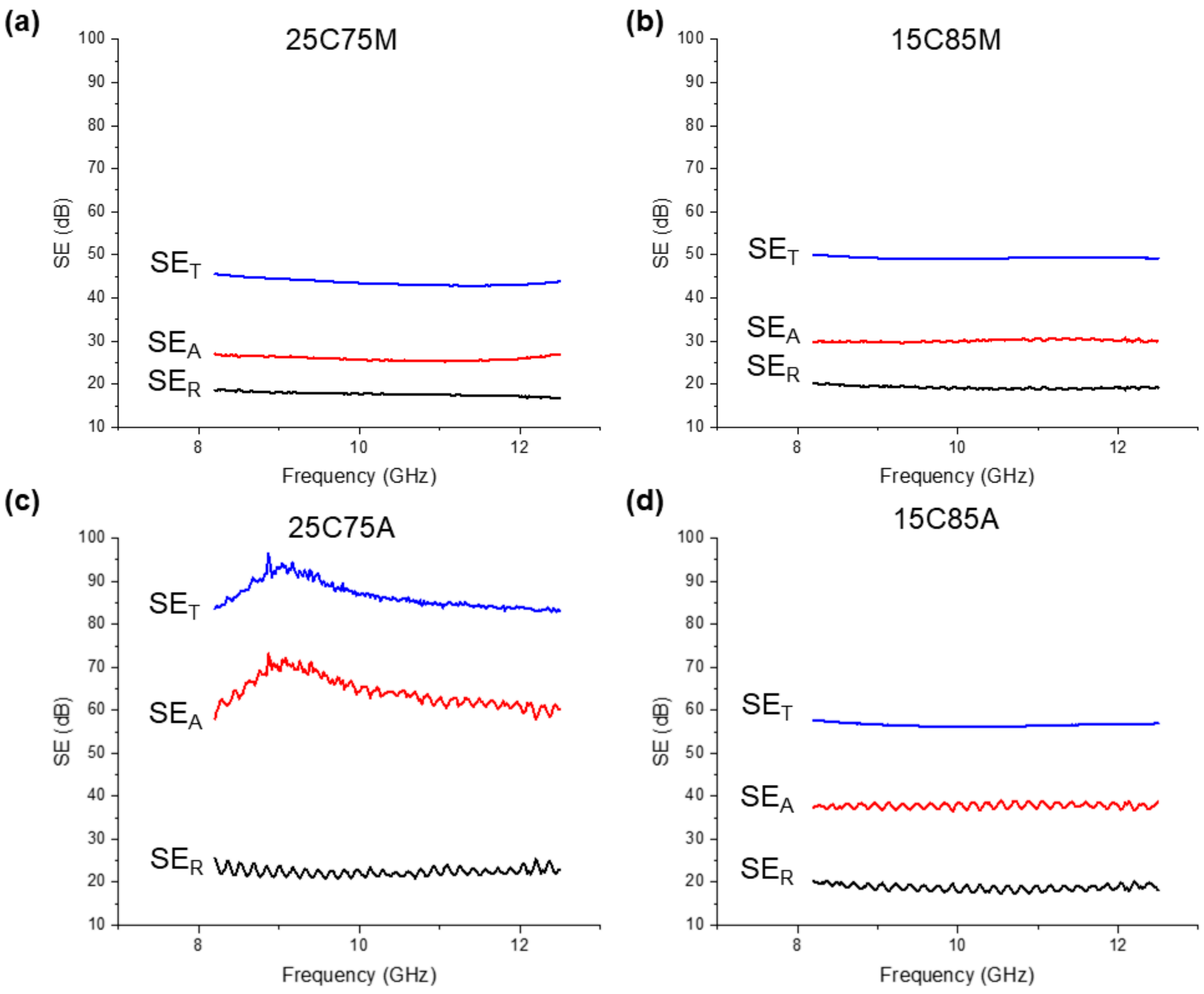

Figure S3. SE_R, SE_A, and SE_T of (a) 25C75M, (b) 15C85M, (c) 25C75A, and (d) 15C85A.

Figure S3 shows reflection and absorption contributions to SE for hybrid sheets containing 75 wt% and 85 wt% filler. Similar to the results in Figure 6(a), all samples show high reflection, at around 99% (20 dB) reflection of wave power. Oscillations in SE_R and SE_A are likely due to small resonances in the transmission lines.
